# Electrodeposition and analysis of thick bismuth films

**DOI:** 10.1038/s41598-023-28042-z

**Published:** 2023-01-21

**Authors:** Kendrich O. Hatfield, Enkeleda Dervishi, Don Johnson, Courtney Clark, Nathan Brown, Genevieve C. Kidman, Darrick J. Williams, Daniel E. Hooks

**Affiliations:** 1 SIGMA-2: Finishing Manufacturing Science, Los Alamos National Laboratories, SM-30 Bikini Atoll Road, Los Alamos, NM 87545 USA; 2 MPA-CINT: Center for Integrated Nanotechnologies, Los Alamos National Laboratories, Los Alamos, NM 87544 USA

**Keywords:** Materials for devices, Materials for energy and catalysis, Structural materials, Techniques and instrumentation

## Abstract

Due to its unique physical and chemical properties, bismuth is an attractive candidate for a wide range of applications such as battery anodes, radiation shielding, and semiconductors, to name a few. This work presents the electrodeposition of mechanically stable and homogenous bismuth films at micron-scale thicknesses. A simple one-step electrodeposition process using either a pulse/reverse or direct current source yielded thick, homogenous, and mechanically stable bismuth films. Morphology, electrochemical behavior, adhesion, and mechanical stability of bismuth coatings plated with varying parameters were characterized via optical profilometry, cyclic voltammetry, electron microscopy, and tribology. Scratch testing on thick electroplated coatings (> 100 µm) revealed similar wear resistance properties between the pulse/reverse plated and direct current electroplated films. This study presents a versatile bismuth electroplating process with the possibility to replace lead in radiation shields with an inexpensive, non-toxic metal, or to make industrially relevant electrocatalytic devices.

## Introduction

Bismuth is a semimetal with interesting physical, electrical, and chemical properties^1,2^. Its unique properties, low toxicity^3^, and availability lead to many applications, such as battery anodes^4^, semiconductors for electrocatalytic degradation of organic waste^5^, and superconductors^6^. Moreover, Bi has a high hydrogen evolution overpotential, allowing higher current efficiency for reductive processes in electrochemical devices, and it has a high electrocatalytic activity toward CO_2_ reduction^7^. Bi is also an effective radiation shielding material^8,9^ and has high magnetoresistance^10^, making it useful in a variety of other applications such as radiation safety and magnetic sensing. Several methods such as sputtering^11^, thermal evaporation^12^, molecular beam epitaxy^13^, and electrodeposition^1,2,14^ have been used to fabricate Bi films. Electrodeposition is particularly attractive, being amenable to mild temperature and pressure conditions on irregularly shaped substrates of a wide range of sizes, with great control over resulting surface morphology^10^. Previous studies have demonstrated electrodeposition of Bi, generally obtaining nanometer^14^ to single micron^1,15^ thicknesses. For some practical applications (particularly radiation shielding), thicker, robust films are desirable^16^. Millimeter-scale electrodeposited Bi coatings have been previously demonstrated a few times in literature on copper films^16^ and a nickel-phosphorous coating^17^ using constant current density deposition methods. However, pulsed electrodeposition is regularly employed to improve coating deposition and brightness^18^, and has been used before for thinner Bi coatings^19^. Possible benefits include a more dense and uniform coating due to the steeper concentration gradient at the surface, as well as better control over film morphology. This work demonstrates a simple, one-step process for depositing Bi films > 100 µm thick with subsequent examination of the effects of pulsed vs direct current plating, differing current densities, and deposition time. Coatings were characterized via electron microscopy, cyclic voltammetry, and tribology to fully understand their structure, adhesion, and mechanical stability.

## Materials and Electrodeposition

### Electrodeposition

Potassium hydroxide (VWR, reagent grade), tartaric acid (Acros organics, 99+%), bismuth (III) nitrate pentahydrate (either Alfa Aesar, 98% or Acros organics, 99.999%), glycerol (VWR, biotechnology grade), and nitric acid (Millipore-Sigma, Emplura, 65%) were used as received for electrodeposition. The plating solution consisted of bismuth nitrate (0.15 M), glycerol (1.4 M), KOH (1.2 M), tartaric acid (0.33 M), and HNO_3_ to adjust pH, which was measured with a Thermo Scientific Orion Star A221 pH meter equipped with a Thermo Scientific 9107BNMD triode. A Dynatronix DuPR10-3-6XR power supply was used with a two-electrode configuration: platinized titanium as an anode/counter electrode (CE) and a gold-plated brass or steel panel (5 µm thick) as the cathode/working electrode. The electrodes were suspended in a glass beaker filled with the plating solution with a magnetic stir bar over a stir plate for all electrodeposition processes. All experiments were performed at room temperature.

### Bismuth film analysis

An Apreo 1 or 2 SEM with an EDAX or Oxford EDS attachment was used for SEM characterization including electron backscatter diffraction (EBSD). Apex OIM software was used to analyze EBSD data. Accelerating voltage for all SEM images was 20 kV. An SP-300 Biologic potentiostat was used with a 3-electrode configuration with a standard calomel reference electrode (saturated KCl), a carbon rod CE, and electrodeposited bismuth working electrode to test hydrogen evolution reaction (HER) on electroplated bismuth in 10% HNO_3_. An RTEC MFT-5000 tribometer was used with a Rockwell type C tip for progressive load scratch testing from 0.1 to 40 N. Prior to tribological tests, Bi films were polished with 10–12 µm (800 grit) sandpaper. A Keyence VK-1000X optical profilometer was used to image films for roughness and assess wear volumes for tribological scratch tests.

XRD was performed on a Rigaku Ultima III diffractometer with fine line sealed Cu tube Kα (λ = 1.5406 Å) x-rays and a D/MAX Ultima series with a maximum power of 3 kW. XRD data was collected on a continuous scan mode in Bragg–Brentano slit geometry over a 2-theta range of 5°–90° with sampling width of 0.05° and scanning speed of 1.5°/min. The divergence slit was set to 2.0 mm, the divergence H.L. slit was set at 10 mm, and the scattering and receiving slits were set to open and open.

## Results and discussion

### Understanding the effects of electrodeposition parameters

**Figure 1:**
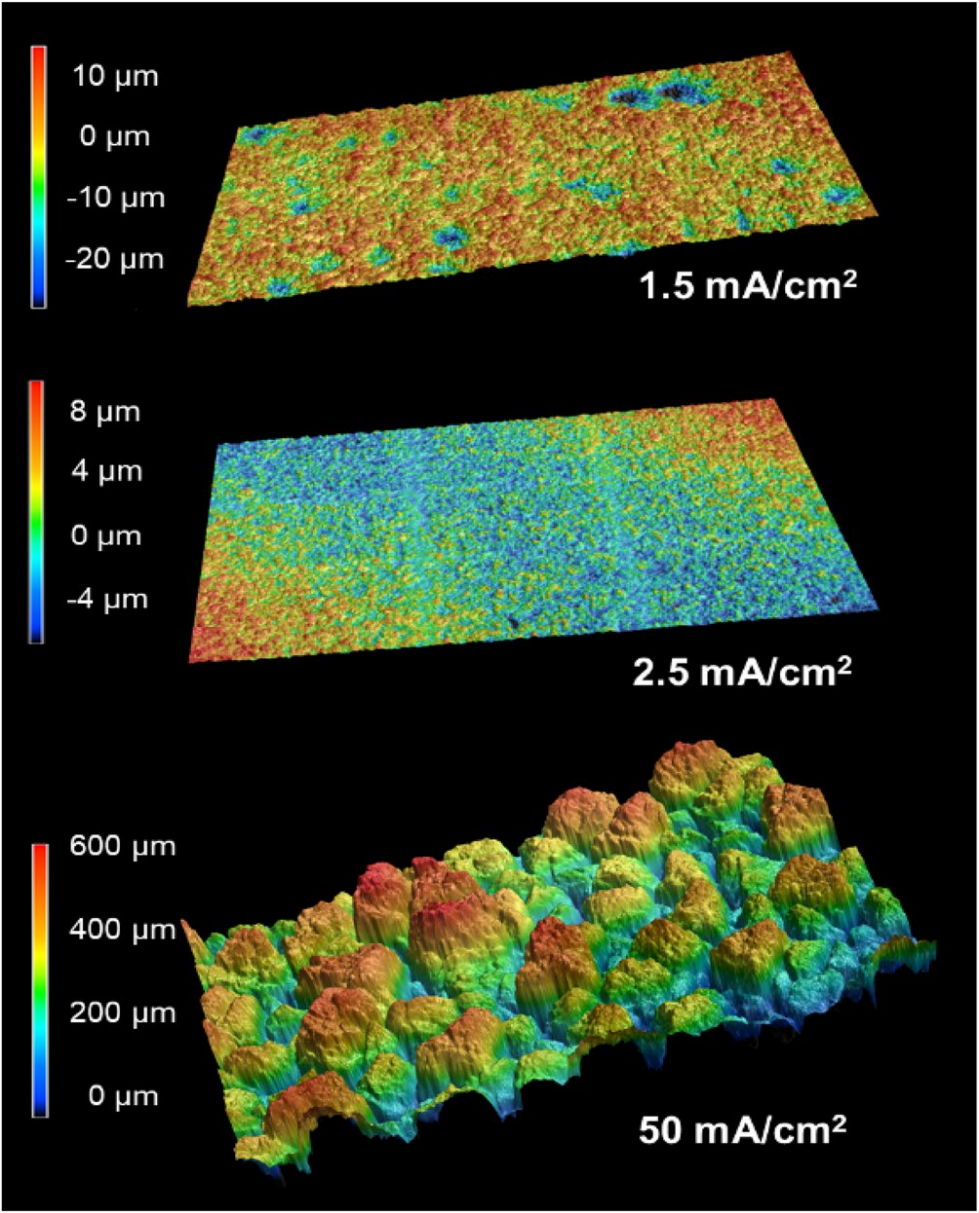
Optical profilometry of intact Bi films electrodeposited at 50 mA/cm^2^, 2.5 mA/cm^2^, and 1.5 mA/cm^2^.

Many parameters profoundly affect Bi electroplating, making proper conditions paramount to obtaining a film with homogenous surface coverage and good adhesion. In addition to Bi(NO_3_)_3_, we added tartaric acid and glycerol as chelating agents in the same manner as Chen et al.^15^ to help moderate film growth^10^ and stabilize Bi^3+^ ion. Plating in this solution is highly sensitive to solution pH, with the best coating obtained at a pH range of 0.01–0.1, resulting in a robust film, while higher pH values produce a film with poor adhesion, able to be wiped away easily by hand. For all further experiments, we adjusted the pH to ~ 0.08 with HNO_3_ before plating. Current density was found to significantly affect film quality; samples plated with current densities of 180 mA/cm^2^ and 50 mA/cm^2^ had inconsistent topographies, with Sa values generally greater than 50 µm and poor adhesion, often delaminating from the substrate upon removal from the electrolyte. Thus, we used a current density of 1.5 mA/cm^2^ for later experiments. Figure 1 shows optical profilometry and photos of intact films grown with direct currents of 50 mA/cm^2^ for 17 h, 2.5 mA/cm^2^ for 24 h, and 1.5 mA/cm^2^ for 24 h. Sandnes et al*.* reported that current densities above 10 mA/cm^2^ resulted in significantly rougher films^1^, agreeing with our results. Current densities of 1.5 mA/cm^2^ (Sa of 5.2 µm) and 2.5 mA/cm^2^ (Sa of 2.6 µm) yielded the brightest and smoothest films.

### Pulsed and direct current electrodeposited bismuth

**Figure 2:**
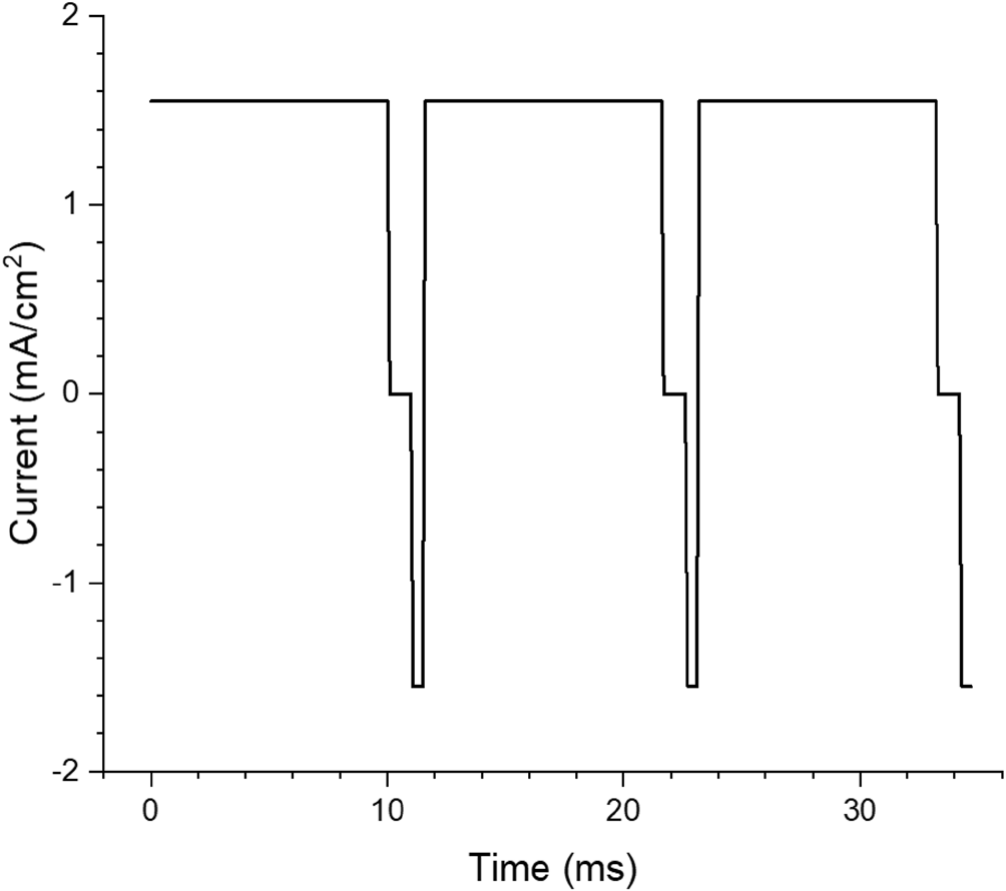
Waveform of the current-controlled pulsed plating process. Both the reverse and forward pulses were 1.5 mA/cm^2^.

Many researchers have used pulsed electrodeposition with a variety of pulse waveforms to obtain more uniform films, higher plating efficiency, and control over morphologies and grain sizes^20^. Pulses used range from sub-millisecond to second timescales and can include “reverse” pulses (i.e. a stripping current) which can improve film uniformity^20^. While a full study of different pulse sequences is beyond the scope of this article, we tested Bi electrodeposition using a pulse/reverse plating process with millisecond-scale pulses (waveform shown in Fig. 2) and compared the resulting coatings with those obtained with direct current plating.

**Figure 3:**
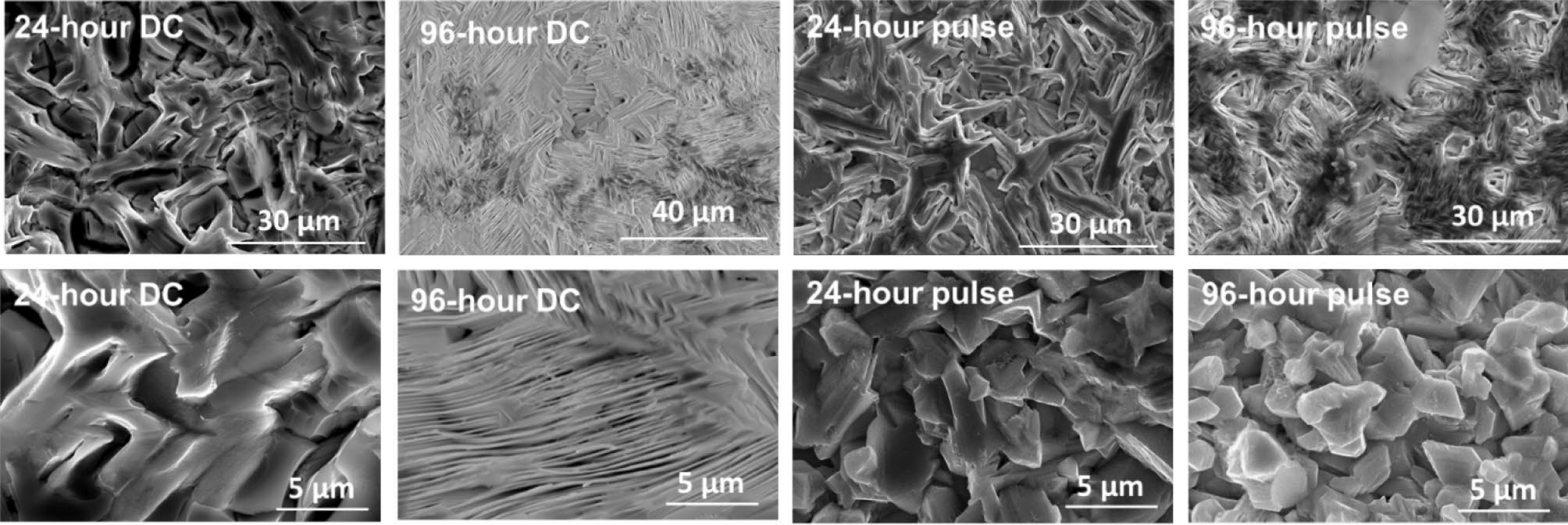
Scanning electron microscopy (20 kV accelerating voltage) of electroplated bismuth at various magnifications.

**Figure 4:**
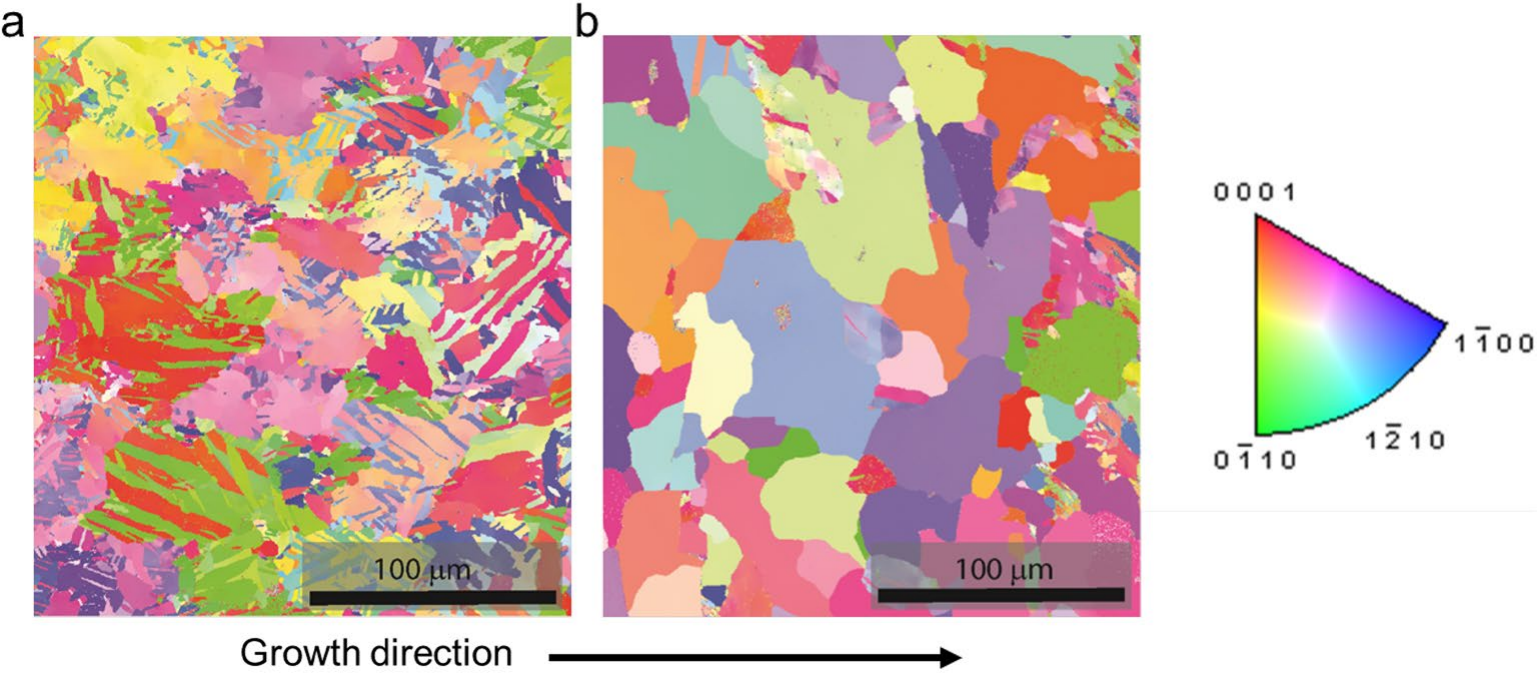
EBSD of 96-h (**a**) DC-plated and (**b**) pulse-plated bismuth coatings.

We used a current density of 1.5 mA/cm^2^ for both pulsed and DC depositions and plated samples for 24 and 96 h. Figure 3 shows SEM images of each sample. The DC-plated samples both show elongated features on the surface, with those on the 96-h DC sample being very thin. On the other hand, the pulse-plated samples displayed a mixed morphology with regions of both elongated features like the 24-h DC sample and “blockier” morphologies with features roughly 2–5 µm in diameter, similar to those found by Gades, et al.^21^. This implies that the electrodeposition waveform has an effect on surface morphology which in turn has been shown to affect electrocatalytic properties^19^. We also observed grains with EBSD on polished cross-sections of 96-h plated samples and estimated grain sizes of 19 µm for the DC-plated coating and 41 µm for the pulse-plated coating (Fig. 4, size histogram in Figure S1). Generally, pulse-plating leads to finer grains than direct current^22^, but our results for the DC-plated grains are likely skewed by the high presence of suspected twinning. Overall, our results indicate that this electrodeposition method offers control over film microstructure, which can significantly impact the physical properties of metals^23^.

We measured film thicknesses (summarized along with deposition rate and deposition efficiency in Table 1) by cross-sectional SEM (Figure S2); thicknesses varied widely for the 24-h pulse/reverse and DC-plated samples, from 80 to 290 µm. As other have previously noted, film thickness is heavily affected by hydrodynamics (i.e. stirring/bath geometry) and cathode placement^24,25^, though the majority of our films were ≥ 100 µm. The 96-h electroplated samples had more consistent thicknesses with DC-plating yielding thicker films than pulsed-plating, likely due to the lower effective current (i.e. duty cycle) of the pulse sequence. These results indicate that either pulsed or DC electroplating is effective to obtain thick (> 100 µm) Bi films with good coverage at high deposition efficiencies (> 70%).

**Table 1:** Electrodeposition results.

Deposition parameters	Coating thickness (µm)	Deposition rate (µm/h)	Deposition efficiency (%)
24-h DC	80–290	3.3–12	98
96-h DC	330–500	3.6–5.1	72
24-h pulse	90–260	4.0–10.6	91
96-h pulse	230–320	2.5–3.1	83

**Figure 5:**
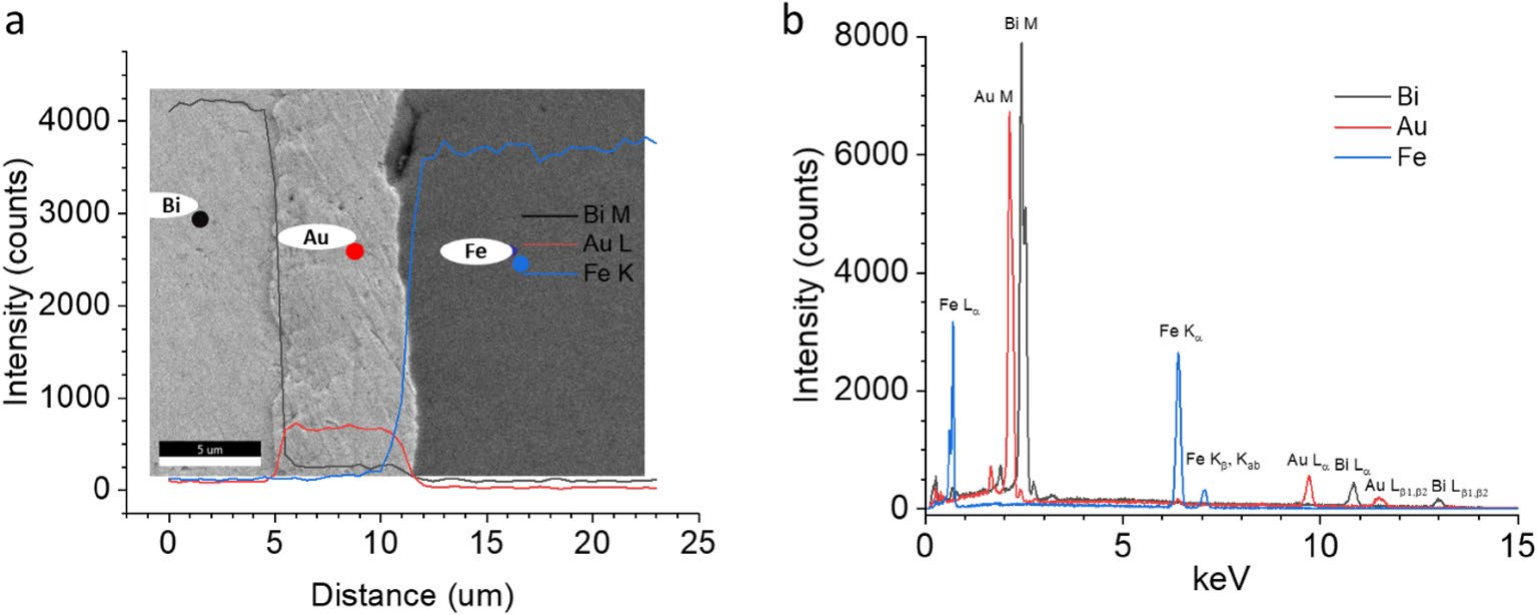
EDS of a bismuth-plated sample cross-section. (**a**) Linescan data across the cross-section from the bismuth M line (black), gold L line (red), and iron K line (blue). (**b**) Overlaid EDS spectra of bismuth (black), gold (red), and iron (blue) areas in the cross section.

An EDS linescan of a Bi-plated cross-section (96-h pulse-plated) shows clear separation between the bismuth, gold, and steel layers on the panel (Fig. 5a). Figure 5b shows overlaid individual EDS spectra of these regions. All samples showed comparable EDS results, shown in Figure S3, and Figure S4 shows an EDS map evidencing a homogenous Bi coverage on the sample surface.

**Figure 6:**
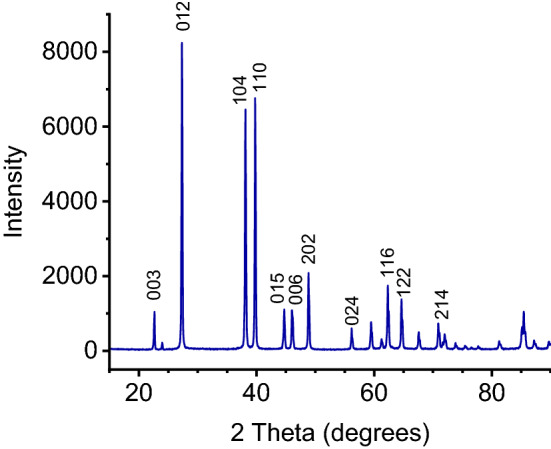
XRD of a polished 24-h pulse-plated Bi coating.

We also performed XRD on a polished Bi surface (24-h pulse-plated) that matches that for Bi (Fig. 6)^26,27^.

### Electrochemical testing

**Figure 7:**
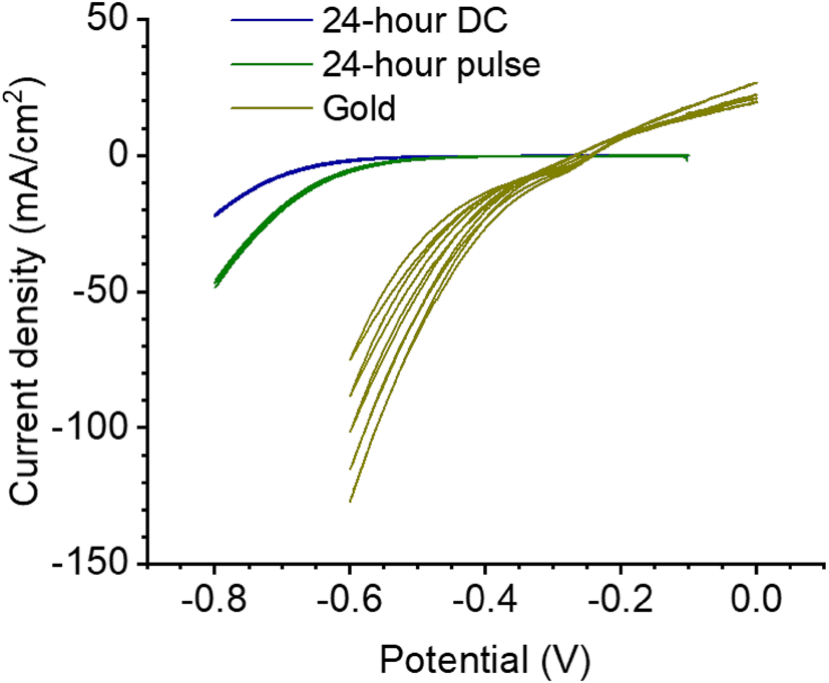
Hydrogen evolution reaction on 24-h pulse- and DC-plated bismuth compared to gold. Cyclic voltammetry performed at 20 mV/s in 10% HNO_3_ using a standard calomel reference electrode (saturated KCL) and a carbon rod counter electrode.

To test the HER activity of electroplated Bi, a critical parameter in electrocatalytic applications, we performed cyclic voltammetry at 20 mV/s on 24-h pulse- and DC-plated bismuth films and plain gold in 10% HNO_3_. Figure 7 shows the higher overpotential of HER on Bi vs Au in the overlaid voltammograms. This is in agreement with Sandnes, et al.^1^, and indicates sufficient coverage of Bi to isolate gold from the solution. The pulse-plated sample yielded lower overpotentials for HER as compared to the DC sample, implying a difference in electrocatalytic activity between the two. This shows promise for electrochemical tunability based on electrodeposition parameters.

### Mechanical properties

**Figure 8:**
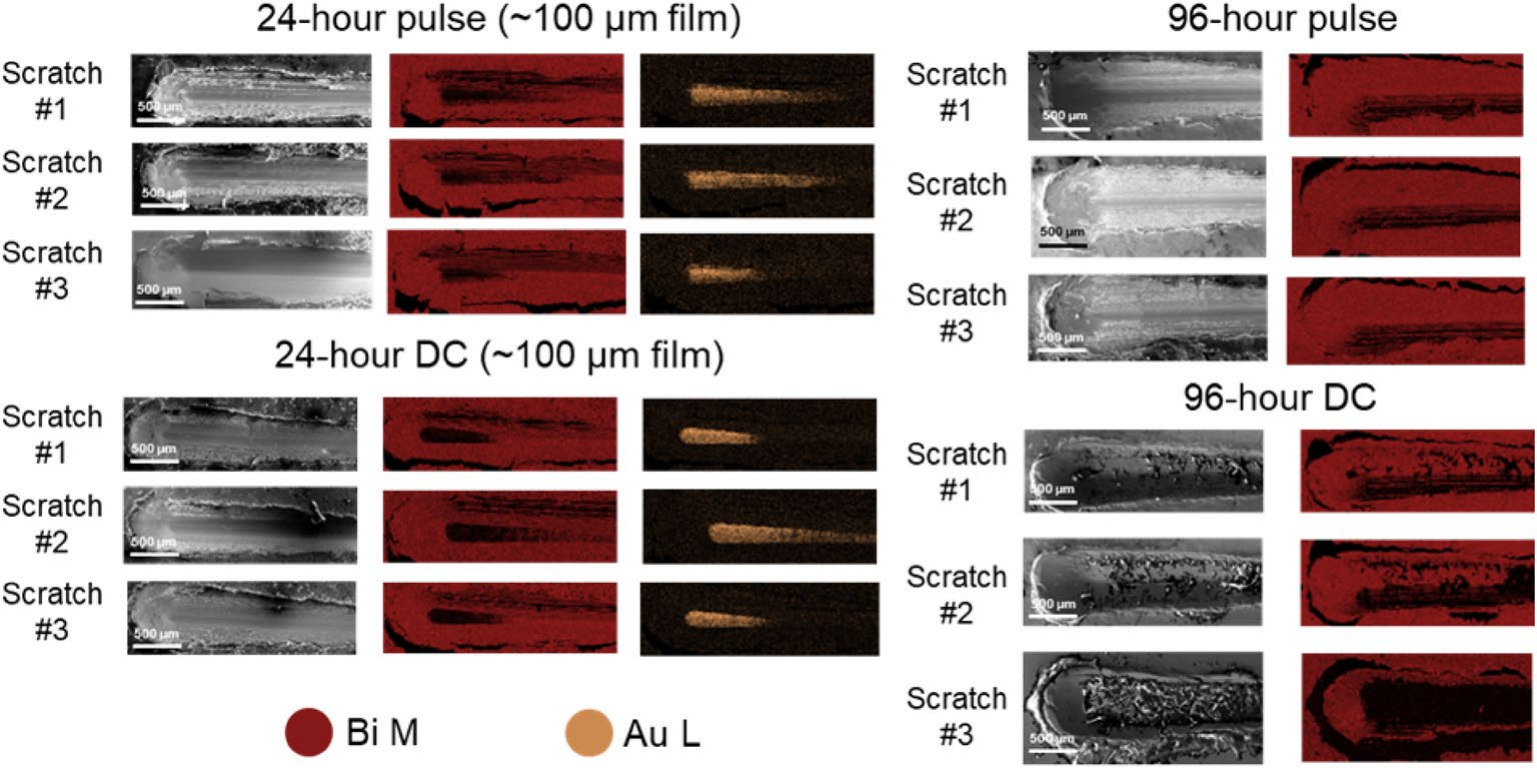
EDS maps of pulse-plated Bi films showing film breakthrough on ~ 100 µm 24-h samples, but not 96-h samples. Maps were made using Bi M lines and Au M lines.

Mechanically robust films are required for practical applications of these films such as radiation shields. We used progressive load scratch testing from 0.1 to 40 N to evaluate tribological performance via the critical load of failure characterized by delamination of the coating from the substrate in the form of gross spalation (Fig. 8-at least 3 scratch tests per sample)^28^. On the thinner 24-h plated samples (~ 100 µm), the scratch stylus broke through the Bi at 25 N for both pulse and DC-plated samples, exposing gold, but without substantial delaminating or cracking of the surrounding bismuth film. Figure 8 demonstrates the EDS maps of the scratch test area for the pulse-plated Bi films at various times. The thicker samples (< 200 µm) withstood up to 40 N (the force limit on our scratch measurement setup) without breaking through to the gold. The wear volume of the scratches, as measured by an optical profilometer, on the 96-h DC plated film were 0.029 ± 0.011 mm^3^, while the wear volume for the 96-h pulse plated sample were 0.035 ± 0.011 mm^3^, indicating similar resistance to scratching between the two plating methods. These results are evidence for good adhesion of the Bi to the Au and robustness for radiation shielding applications regardless of pulse waveform.

## Conclusions

In this study, we demonstrated a simple, non-toxic process for electrodepositing thick (> 100 µm) Bi films on gold substrates and evaluated the effects of deposition time and pulsed vs DC electroplating. Increasing deposition times with both constant current and pulse/reverse methods lead to thicker films, showing potential for industrially usable, robust films for radioactive shielding applications. EDS showed a relatively pure and homogenous distribution of Bi throughout the film regardless of deposition parameters with a current density of 1.5 mA/cm^2^. Pulsed electrodeposition impacts surface morphology, grain size, and electrocatalytic activity of the electrolyte. Cyclic voltammetry showed higher HER activity on a pulse-plated sample compared to a DC-plated coating, implying a tunability for practical electrochemical applications. Mechanical strengths of DC- and pulse-plated coatings were similar, with scratch testing showing complete breakthrough of thin 24-h plated samples at 25 N with a Rockwell tip without excessive cracking or delamination. Scratch testing on samples > 200 µm also revealed similar wear resistance properties between DC and pulse plated films. Due to the versatility of electroplating toward substrates of irregular shapes and sizes, this study demonstrates a practical method of replacing lead in radiation shields with an inexpensive, non-toxic metal or for making industrially relevant electrocatalytic devices. Future experiments could involve testing films of varying thicknesses in a radioactive shielding environment or for carbon dioxide reduction to evaluate optimal Bi coating parameters for these applications.

## Supplementary Information

Supplementary Figures.

## Data Availability

All data generated or analyses during this study are included in this published article [and its supplementary information files].
