# Supplementary material for: Electrodeposition and analysis of thick bismuth films
Source: Sci Rep. 2023 Jan 21;13:1202. doi: 10.1038/s41598-023-28042-z (PMC9867696; doi:10.1038/s41598-023-28042-z)
Supplement: Supplementary file 1 — Supplementary Figures. [file 41598_2023_28042_MOESM1_ESM.docx]

**Electrodeposition and Analysis of Thick Bismuth Films**

Supplemental Information

Kendrich O. Hatfield^1^, Enkeleda Dervishi^1*^, Don Johnson^1^, Courtney Clark^1^, Nathan Brown^1^, Genevieve C. Kidman^1^, Darrick J. Williams^2^, and Daniel E. Hooks^1,2^

^1^SIGMA-2: Finishing Manufacturing Science, and ^2^MPA-CINT: Center for Integrated Nanotechnologies, Los Alamos National Laboratories, Los Alamos, New Mexico, 87544.


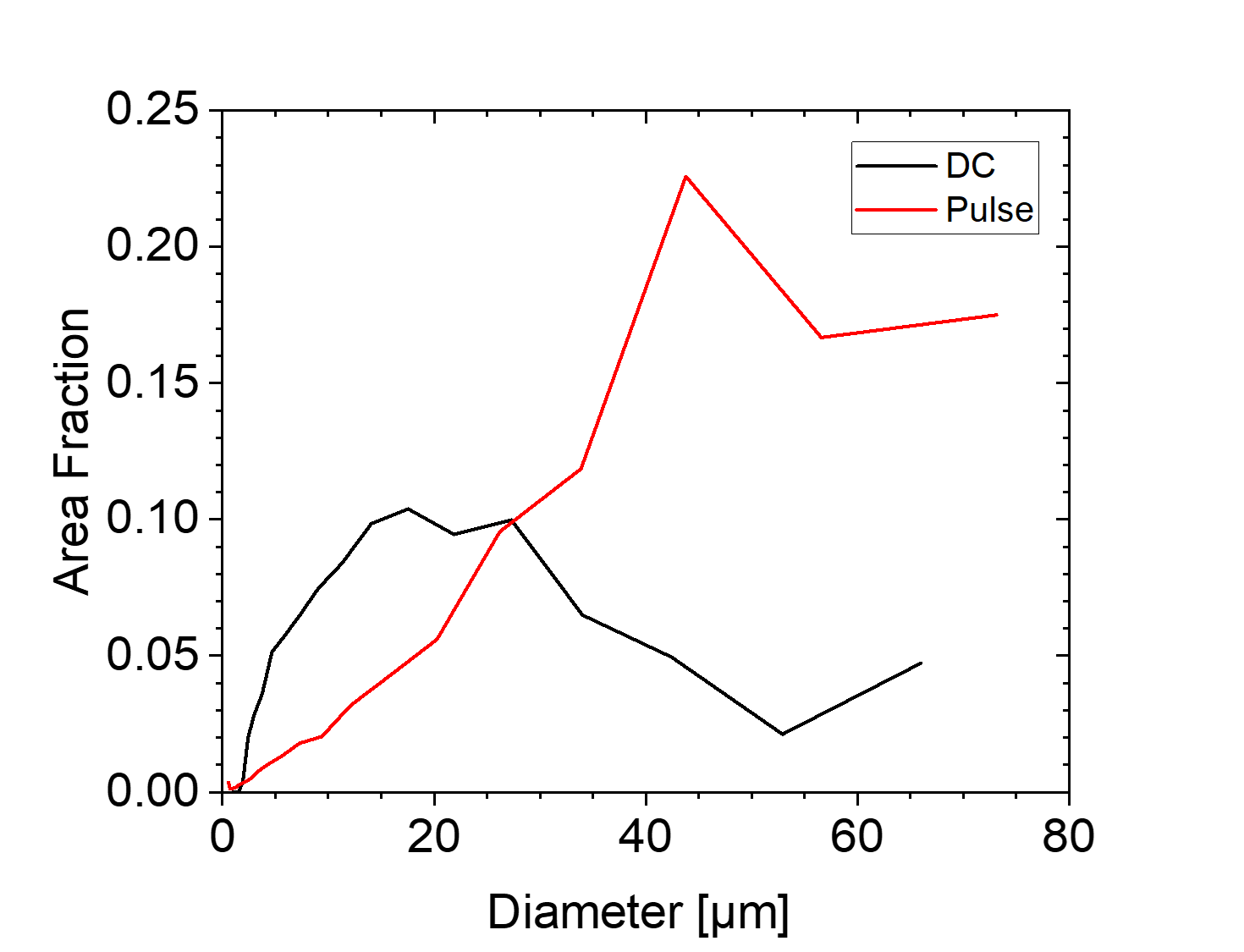


**Figure S1:** Histogram of grain sizes of 96-hour electroplated bismuth coating cross-sections.


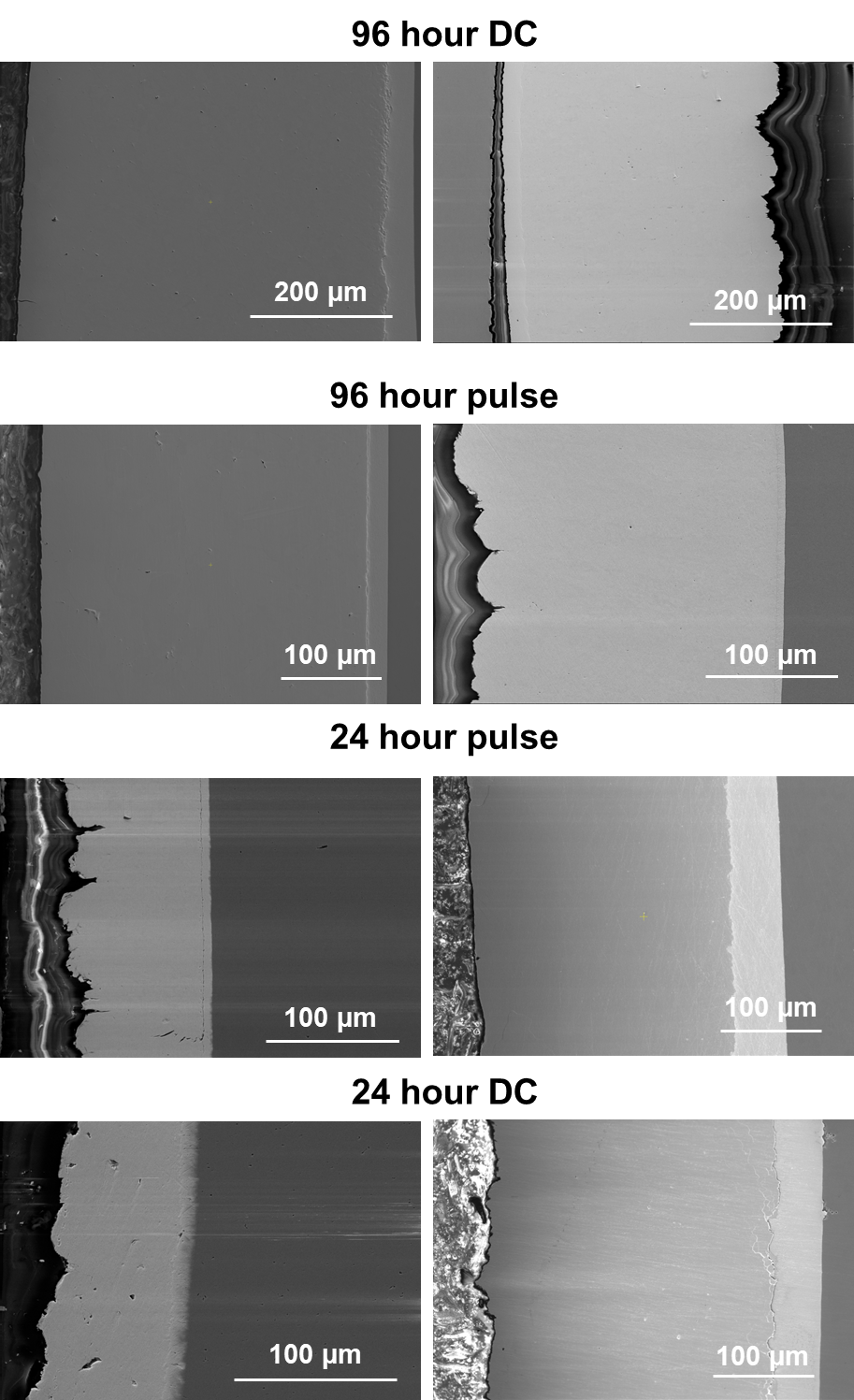


**Figure S2:** Example cross-sectional SEM of electroplated Bi films used to measure film thickness.

**Figure S3:** Cross-sectional EDS of Bi films, including line scans and a map of a 96-hour pulse-plated sample. Note there was some delamination of the Au from the steel substrate in the 96-hour DC-plated cross-section, apparent in the SEM image and EDS linescan.


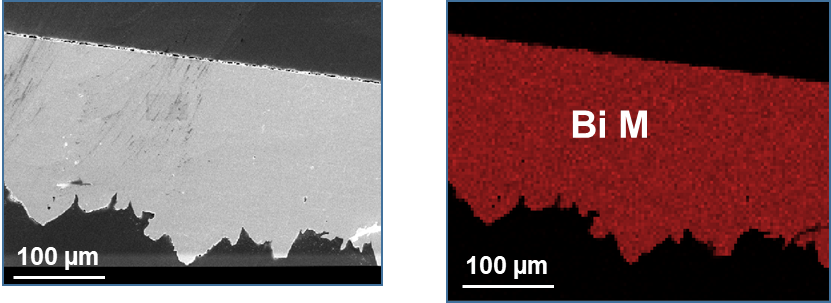

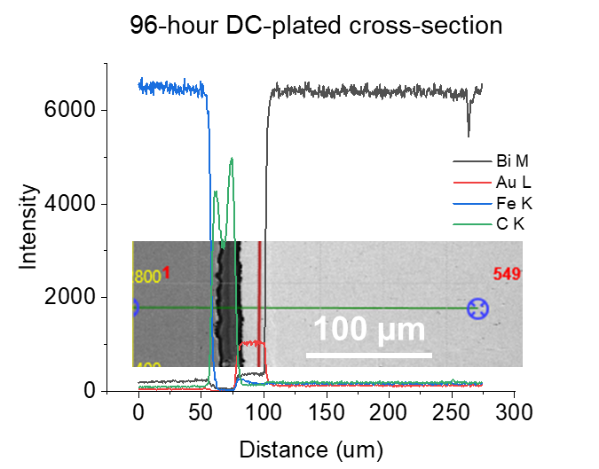

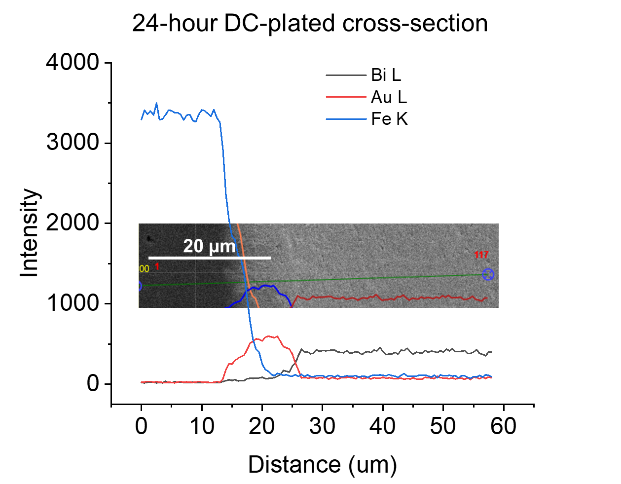

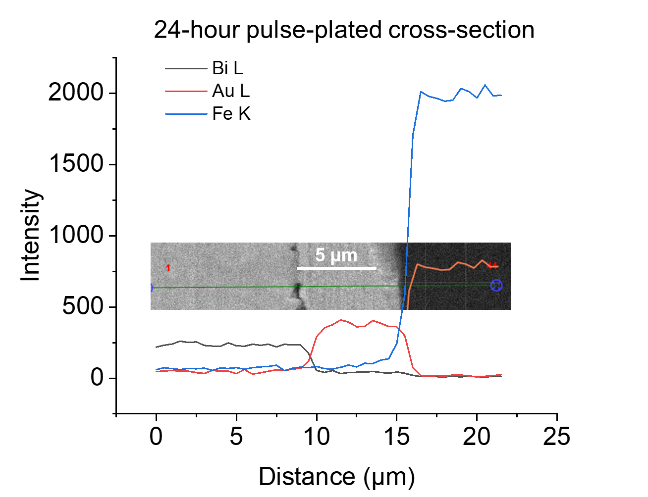

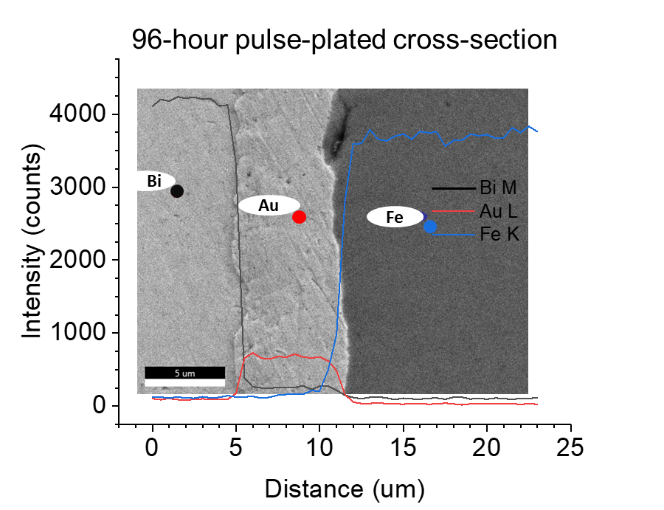

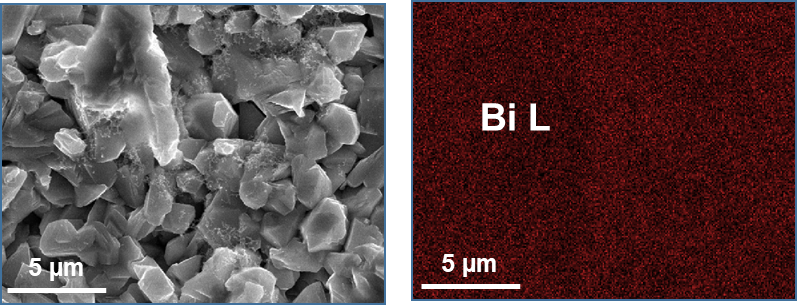


**Figure S4:** EDS map of a 96-hour pulse-plated sample showing homogenous coverage of Bi using the L line.
